# Understanding the Unique Microenvironment in the Aging Liver

**DOI:** 10.3389/fmed.2022.842024

**Published:** 2022-02-24

**Authors:** Yalei Zhao, Ya Yang, Qian Li, Jianzhou Li

**Affiliations:** ^1^Department of Infectious Diseases, The First Affiliated Hospital of Xi'an Jiaotong University, Xi'an, China; ^2^State Key Laboratory for Diagnosis and Treatment of Infectious Diseases, National Clinical Research Center for Infectious Diseases, Collaborative Innovation Center for Diagnosis and Treatment of Infectious Diseases, College of Medicine, The First Affiliated Hospital, Zhejiang University, Hangzhou, China

**Keywords:** aging, microenvironment, regeneration, hepatic cells, extracellular matrix

## Abstract

In the past decades, many studies have focused on aging because of our pursuit of longevity. With lifespans extended, the regenerative capacity of the liver gradually declines due to the existence of aging. This is partially due to the unique microenvironment in the aged liver, which affects a series of physiological processes. In this review, we summarize the related researches in the last decade and try to highlight the aging-related alterations in the aged liver.

## Introduction

During aging, the liver undergoes a series of degenerative changes. Briefly, it presents a progressive decrease in functional liver mass, thus reducing its functional reserve, making it more difficult to maintain homeostasis and vulnerable to external stress or damage (1). Till now, the mechanisms underlying liver aging still remain unclear. As we known, the main causes of aging are DNA damage, telomeres shortening, epigenic alterations, and impairment of proteostasis (2). The aged liver is usually accompanied with failure of regeneration, metabolic dysfunction, redox imbalance, and development of chronic or malignant liver diseases (3–6). The impairment of regenerative capacity in the aged liver is affected by both intracellular factors and extracellular factors (7). Intriguingly, we may be able to recover their regenerative capacity via changing a microenvironment for the senescent hepatocytes (8). The aging-related alterations in the liver form a unique microenvironment and affect a series of physiological processes. Moreover, this unique microenvironment may function as a vital role that causes the liver to become susceptible to chronic diseases or tumors (9). For instance, it affects the fate of hepatocytes and promotes neoplastic development (10). Moreover, hepatocytes in this microenvironment are more susceptible to ischemia/reperfusion (I/R) injury (11–13). Of particular interest is the way to effectively eliminate the effects of aging and reverse the unique aging microenvironment in the aged liver. As reviewed by Xu et al., modulation of autophagy could function as an effective strategy for reverse of the aged liver (14). Autophagy mainly functions as a cytoprective role in liver diseases. Modulation of autophagy could markedly alleviate aging-related liver injury, promote liver regeneration, block I/R induced injury, and reverse the aging microenvironment in the aged liver (14–17).

## Hallmarks of Hepatic Aging Process

**Figure 1:**
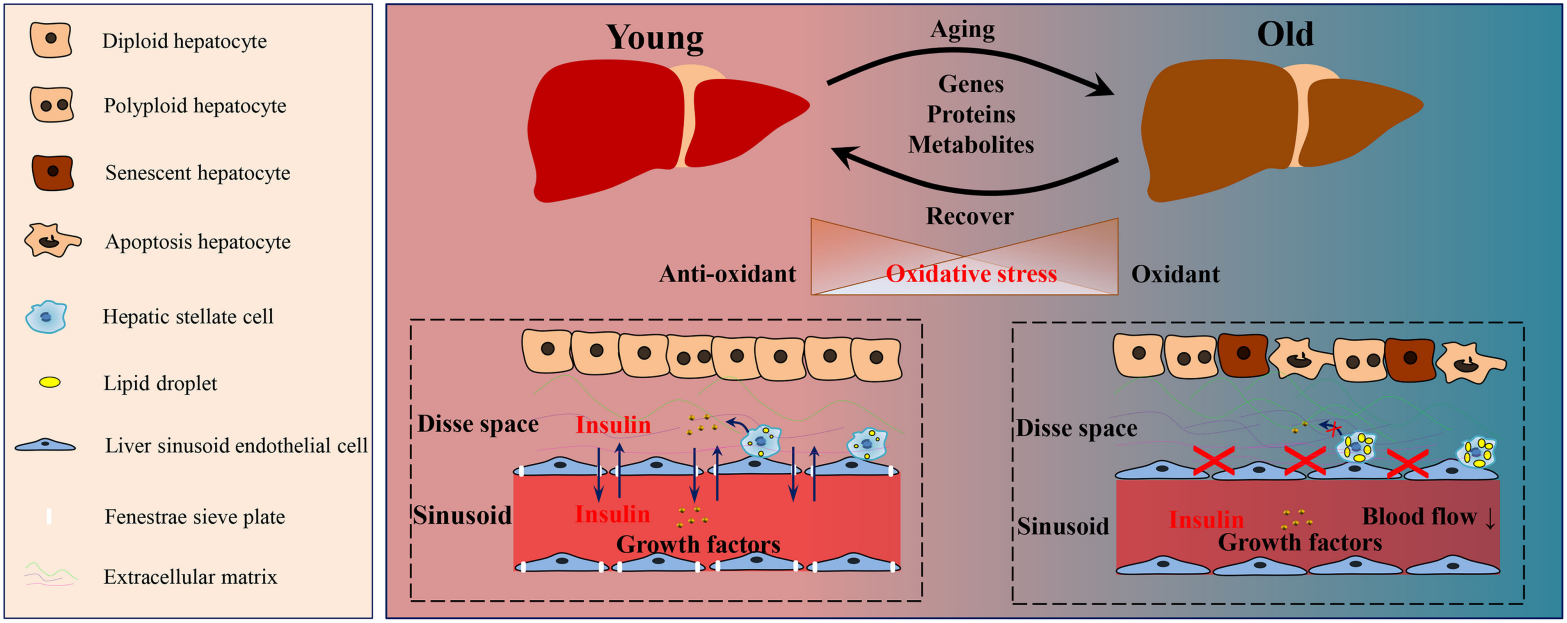
Schematic diagram of the aging-related changes in the liver. During the aging process of the liver, a series of alterations was observed in genes, proteins and metabolites. The oxidative homeostasis was disrupted, thus lead to oxidative stress. During aging, the number of polyploid, senescent, and apoptotic hepatocytes increased. Hepatic stellate cells in the aged liver contained more lipid droplets and released less growth factors than those in the young liver. The defenestration liver sinusoid endothelial cells occurred during aging, and thus disrupted the substance exchange, such as insulin and growth factors. Moreover, more extracellular matrix was deposited in Disse space in the aged liver.

Over the past years, our understanding of the aging process experiences a great advance. Of notable interest is calling for accurate hallmarks of aging in different organisms and in different organs. In 2013, Lopez-Otin et al. reviewed and concluded 9 cellular hallmarks of aging: genomic instability, epigenetic alterations, loss of proteostasis, telomere attrition, mitochondrial dysfunction, deregulated nutrient sensing, cellular senescence, stem cell exhaustion, and altered intercellular communication (18). Aging related changes in the aged liver occur at genomic, epigenetic, molecular, cellular and sub-cellular levels. Moreover, the aging process in the liver undergoes all the cellular hallmarks, these changes occur in various cells in the liver (parenchymal cells and non-parenchymal cells) and extracellular matrix (ECM) (Figure 1), and finally lead to impairment of liver function (19–21). In a recently published review, Hunt et al. enumerated the hallmarks of the aging process in the liver. Besides the hallmarks reviewed by Lopez-Otin et al., they also covered response to endoplasmic reticulum (ER) stress and aging human liver into discussion (18). Recent years, long non-coding RNAs and microRNAs have gained a lot of interest, and they are considered to be involved in the aging process, especially in the metabolic modulation and intercellular communication (22–25). Moreover, Barbosa et al. also highlighted the relevance of autophagy activity to the hallmarks of aging (26).

## Aging Liver: Suitable Animal Models

To fully understand the aging-related alterations in liver, suitable animal models are deemed necessary. Generally, these animal models can be divided into: naturally aging models, accelerated aging models, mimetic aging models, and transgenic aging models. Naturally aging models, especially aging rodents (mice aged ≥ 12 months, rats aged ≥ 16 months), are widely used in aging research because of their similarity of characteristics with human aging process. They have been applied in most of the studies related to aging liver in the last decade. Moreover, researchers used the senescence-accelerated mouse (SAMP10) to study the underlying mechanism of the aging liver (27, 28). In addition, mimetic aging models are also widely applied, which included D-galactose, radiation, β-amyloid, aluminum chloride induced aging models. Of these, D-galactose mimics naturally aging via inducing mitochondrial dysfunction and oxidative stress, increasing inflammation and apoptosis in the liver. Radiation induced aging models are usually used in cancer treatment (radiotherapy) related aging researches (29). Application of β-amyloid and aluminum chloride induces brain disorders, and these models are usually applied in Alzheimer's Disease related studies (30, 31). In recent years, transgenic aging models gain more and more attention with the development of gene editing technologies. Nevertheless, these animal models can only partially reproduce the typical pathological and biochemical alterations of aging.

## Aging Related Alterations in the Liver

Currently, many researchers have reported the mechanisms of aging and its effects on the liver. In 2013, Capel et al. found that aging-related genes in the aged liver were mainly related to xenobiotic and fatty acid metabolism, retinoid X receptor function, and oxidative stress (32). In another microarray based study in the liver of aged rats, various genes involved in lipid metabolism and cell growth were downregulated (33). In 2015, White et al. revealed the main aging related alterations were involved in immune response, metabolic processes, RNA modification, and cell activation (34). Bochkis et al. showed that the inflammatory regulators (NFκB, IRF3, TLR4, et al.) upregulated related genes in the aged livers, nucleosome occupancy increased with age in mammalian liver (35). Sato et al. revealed that the aging process induced reprogramming of circadian rhythms in the liver. They showed that hepatic metabolic processes, such as protein acetylation and Nicotinamide adenine dinucleotide (NAD+) metabolism, were critically involved in the circadian change and remodel (36). In 2019, Chen et al. reported the transcriptional profiling of aging liver in Yana pigs (37). They showed that the up-regulated genes in pig aging liver were mainly involved in immune response, while the down-regulated genes were mainly enriched for metabolism (34). In another research, Bacalini et al. identified aging correlated genes enriched in the following biological processes: inflammatory response, interferon gamma response, and allograft rejection (38).

It is well known that oxidative stress plays a key role in the process of aging. Considering mitochondria and peroxisomes are important sources of reactive oxygen species (ROS), several studies have characterized aging related changes in liver peroxisomes and mitochondria using proteomics technology. In 2011, Amelina et al. identified 8 differentially expressed proteins of young (10-week-old) vs. old (18-month-old) in mouse liver peroxisomes (39). Five proteins were significantly up-regulated in the aged liver: epoxide hydrolase 2, 3-ketoacyl-CoA thiolase A, peroxisomal sarcosine oxidase, peroxisomal 2,4-dienoyl-CoA reductase, and prohibitin-2. And 3 proteins were down-regulated: pancreatic alpha-amylase, cytochrome c oxidase subunit 6C, and Cytochrome b-c1 complex subunit 2. These differentially expressed proteins mainly involved in the following processes: detoxification of xenobiotics, peroxisomal-oxidation, and production of ROS (39). In 2011, Musicco et al. identified differentially expressed hepatic mitochondrial proteins between old (28-month-old) and adult (12-month-old) rats. These differentially expressed proteins were involved in several mitochondrial metabolic pathways, such as pyruvate dehydrogenase complex, tricarboxylic acid cycle, and oxidative phosphorylation system chain (40). In another study, Bakala et al. revealed 16 differentially expressed hepatic mitochondrial proteins between young (3-month-old) and old (20-month-old) rats. They also found that most of these proteins were enzymes related to metabolic processes including fatty acid β-oxidation reaction, oxidative phosphorylation system chain complex I/V components, and tricarboxylic acid cycle (41). Moreover, to deeply reveal the aging-related organ specific function deterioration, Ori et al. identified the molecular alterations in the liver in young (6-month-old) and old (24-month-old) rats using an integrated approach (42). In this study, the researchers revealed several aged-related changes: altered translation and protein abundance, protein re-localization, alternative splicing, and altered protein phosphorylation. The liver proteome homeostasis was affected during aging, and the majority of these alterations were due to transcriptional regulation. The age-specific impacts on the liver were tightly related to the liver function: small molecule metabolism, monosaccharide metabolism, angiogenesis, protein degradation, complement activation, and antigen processing (42). To further investigate the molecular networks of longevity, Heinze et al. conducted a cross-species comparison between the long-lived naked mole-rats and shorter-lived guinea pigs (43). They revealed that 30 hepatic proteins were associated with sustaining longevity and most of these proteins were linked to lipid or fatty acid, and xenobiotic metabolism (43). Taken together, all of the above studies advance our understanding of aging-related changes in liver proteins that are significantly associated with organ function. However, we have to recognize there are several limitations to these studies. Firstly, different animal models are utilized in these studies. Some researchers choose mice as animal models, while others choose rats. In addition, they selected animals of different ages for the aging study. Secondly, the number of biological replicates is limited. Thus, these above findings need to be further validated.

In addition, metabolites are markedly altered during aging. Metabolomics technology enables us to fully understand the dynamic changes of metabolites, qualitatively and quantitatively (44). In 2013, Smiljanic et al. revealed aging-related cholesterol metabolism alterations in the liver of aged rats, and found that aging induced elevation of 2 cholesterol precursors: lanosterol and lathosterol (45). In another study, Pagliassotti et al. identified several metabolites that reduced in the liver of aged mice: pyruvate, lactate, nicotinamide, UDP-N-acetylglucosamine and UDP-N-acetylgalactosamine (46). Of these, UDP-N-acetylglucosamine was associated with longevity and protein quality control (47). Sato et al. quantitatively measured NAD+ and metabolites participated in its biosynthetic pathway (36). The authors revealed that nicotinamide and ADP ribose were significantly altered during aging and identified NAD+ metabolism as a core liver specific circadian metabolic pathway of aging. The improvement of NAD+ availability increased sirtuin 1 activity and rescued protein acetylation, thereby reversed the aging process (36). In 2019, Ando et al. found that ether-linked diacylglycerols accumulated in the liver of aged mice, which may associate with cell arrest and apoptosis (48). Wesley et al. reported the liver specific elevation of B-alanine and uridine, and the decrease of NAD and formate in aged rats (49).

### Hepatic Cells of the Aging Liver

In the aged liver, number of hepatocytes decreases, and remnant hepatocytes experience an autonomous decline in the regenerative capacity (50). Moreover, the aging related hepatic structural changes form a hypoxia condition, which induces higher glucose production and elevation of phosphoenol pyruvate carboxykinase, a gluconeogenesis-regulating enzyme. This process is regulated by hepatocyte nuclear factor 4 α, and targeting its expression can reverse the aging related effects on gluconeogenesis (51). In addition, apoptotic, senescent and polyploidy hepatocytes accumulate in the aged liver (52–54). Intriguingly, targeted approaches that modulating the level of apoptosis can block the aging effects (52). The senescent hepatocytes are typically characterized by accumulation of DNA damage, activation of tumor suppressor pathways (p53, p16ink4a, and C/EBPα) (53, 55, 56). These senescent cells remain metabolically active, but no longer able to proliferate. They present with the senescence-associated secretory phenotype and affect their neighboring cells, e.g., induce macrophage migration, immune cell recruitment, and modulation of ECM (52, 57). For polyploidy hepatocytes, its role in aging liver still remains controversial. Polyploidy is also considered as a feature of the aging process, and several hypotheses have been proposed to explain their function and significance (58, 59). Some studies indicated that hepatocyte polyploidy is a terminally differentiated state (58). It is suggested that the polyploidy state restricted proliferation of hepatocytes (60). Reversal of hepatocyte ploidy can reverse the aging-related function disorder (61). Oppositely, other studies reported that hepatocyte polyploidy may enhance liver function, retains the ability to divide and proliferate, and even contributes to hepatocyte turnover during aging (58, 62, 63). Verma et al. found that hepatocytes and cholangiocytes in liver donors over a wide range showed no telomere shortening (64). However, Aini et al. observed telomere shortening in hepatocytes in human liver allografts (54). Thus, it is still debated about the contribution of telomere shortening to senescent hepatocytes (20). Intriguingly, Lin et al. identified a novel subset of hepatocytes with high levels of telomerase that repopulated the liver (65). This emerging view may enable us to re-consider the role of telomerase in senescent hepatocytes. Moreover, continuous cell proliferation could reactive telomerase, reverse senescence and polyploid in hepatocytes during aging (53). In addition, Bacalini et al. reported DNA methylation of 6 age-related differentially methylated positions (ELOVL2 island, MACROD1 island, CYP1B1 island, CCNJ island, ZIC1 island, ZIC1 shore) in primary human hepatocytes (38). IFN-α and its related signaling elevate in aging hepatocytes (66). During aging, hepatocytes highly express activin A and p15INK4b, which are thought related to decreased proliferation and increased apoptosis (67). Ultrastructural changes of aged hepatocytes were characterized by cytoplasmic vacuolization, hypertrophy, and changes in density of the nuclei and mitochondria (68). Moreover, somatic mutations were accumulated with age in human hepatocytes (69). However, post-translational modification of histone (H3K9me3/H3K14ac modification) was decreased, which subsequently lead to gene alteration (70). Liver sinusoidal endothelial cells (LESCs) are critically involved in managing the liver microenvironment. However, LSECs in the aged liver are gradually thickened and undergo typical age-related changes (defenestration or pseudocapillarization) (71–73). Pseudocapillarization of the sinusoid lining delays liver regeneration in the aged liver via disturbing endothelium-dependent processes, decreasing hepatosinusoidal blood flow, impairing platelet adhesion to sinusoid, and preventing growth factors to reach target cells (74). The old LESCs present with reduced endocytic capacity and low responsive to 2,5-Dimethoxy-4-Iodoamphetamine (71, 75). In addition, aging-related changes in LESCs lead to hepatic insulin resistance and influence glucose homeostasis, consistent with the reduced transendothelial transfer activity of insulin (76). Moreover, aging LESCs leads to up-regulation of cell adhesion molecules, thus induces an accumulation of CD68 macrophages and neutrophils. Together with the up-regulation of p16 and fibrinogen 2, these alterations induced by aging LESCs finally lead to pro-inflammatory phenotype (77). However, these changes may be reversed by targeting aging-related defenestration (78). HSCs reside around LESCs and their activation is partially regulated by LESCs (79). Thus, the above mentioned aging-related LESCs changes also have an impact on HSCs. In general, HSCs are activated and transdifferentiate into myofibroblasts (α-SMA+) upon injury. Aging is related to the hyperplasia of HSCs. During aging, it exists an increase in the number and diameter of lipid droplets in HSCs (80). Telomere attrition, a hallmark of the aging liver, also occurs in HSCs (64). In addition, integrin α5/β1 decreases in HSCs and thereby reduces the levels of hepatocyte growth factor in the aged liver, thus impairs liver regeneration (81). Moreover, production of ECM components by HSCs, such as laminin, is impaired in aged mice (81, 82). Thus, aging-related HSCs alterations have a vital influence on ECM remodeling. As we know, there is a close association between HSCs and liver progenitor cells (LPCs) (83), and HSCs may act as positive regulators of LPCs. LPCs, or called oval cells, are critically involved in maintaining the liver homeostasis. However, they are rarely observed in normal conditions. LPCs activation exists in various liver diseases, such as viral hepatitis, liver cirrhosis, and liver cancer (84, 85). Upon injury, LPCs activate, proliferate, and accumulate around the portal vein or central vein, and this process is termed ductual reaction (86). However, their activation decreases with age. As reported, Thy-1 (+) LPCs declined in aged donor, which might responsible for impaired liver regeneration (87). Qian et al. revealed that oval cell in aged mice showed low response to 3,5-diethoxycarbonyl-1,4-dihydrocollidine induced liver injury, due to the down-regulation of laminin (82).

Besides non-immune cells mentioned above, immune cells are also play important roles in maintaining liver function. Aged liver exhibits increased immune cell infiltration, such as macrophages, T-cells, B-cells, NK cells, and neutrophils, which is accompanied by a high inflammatory status (88). The increased immune cell infiltration and high inflammatory status in the aged liver were thought to be a detrimental factor of liver regeneration. Till now, our understanding of the infiltrated immune cells in aged liver is still superficial. During aging, neutrophils are recruited by p16Ink4a-expressing cells and induce oxidative damage telomeres in non-immune cells (89). Intriguingly, modulation of the immune cell infiltration in aged liver, such as depletion of macrophages and NK cells could significantly leads to improved regeneration (66). Nevertheless, aging leads to decline of macrophage function. Briefly, aging leads to impaired autophagy and phagocytosis, dysregulated secretion of pro-inflammatory mediators, alterations in cell morphology and distribution, and changes in epigenetic signature (90).

### Extracellular Matrix of the Aging Liver

The ECM provides structural support and reside environment for liver cells. In recent years, the ECM gains a lot of attention and some of its novel functions in regenerative medicine have been uncovered (91, 92). The role of the ECM in aging and longevity has been reviewed (93, 94). Major aging-induced modifications of the ECM are glycation, fragmentation and carbamylation (94). Karsdal et al. outlined the aging-related changes in the turnover of the ECM by detecting 15 serum biomarkers, which provided a new perspective for the therapy of chronic liver disease (95, 96). Mahmoud et al. observed increased ECM deposit in the Disse space in the livers of old rats by transmission electron microscopy (68). Delire et al. showed that the liver of old mice with impaired ECM remodeling capacity was more susceptible to carbon tetrachloride induced fibrosis, owing to a reduction of the chemokine (C-X-C motif) ligand 9 and matrix metallo-proteinases-13 axis (19).

Till now, the researches referred to the aging-related alterations of liver ECM are limited. The ECM is important for not only structural support, and also for maintaining liver function during aging. Aging induces impairment of ECM remodeling, making the liver more susceptible to injury. However, to fully understand the role of the ECM in the aging liver, we still need more experimental work.

## Concluding Remarks and Future Directions

Liver aging is a complicated process with a series of organ-specific alterations, from genes, proteins to metabolites. These alterations form a unique microenvironment in the aged liver and have an impact on liver functions. Elimination of the aging effects and reversal of the aging microenvironment are beneficial to the reparative capacity of the aged liver. In the future, the development of drugs or anti-aging vaccines to treat aging-related liver disorders will be urgently needed. Modulation of autophagy is considered as a promising “rejuvenation” strategy for the aging liver. However, how to effectively and appropriately modulate autophagy without harm is still not available. Meanwhile, novel therapeutic targets for reversing the hepatic aging microenvironment are hotly pursued. Hopefully, we will develop effective anti-aging strategies based on novel therapeutic targets in the future.

## Author Contributions

JL and YZ designed the work. YZ, YY, and QL searched the databases and listed the related literature. YZ and YY drafted the manuscript. JL provided critical revision of the manuscript. All authors approved the final version.

## Funding

We thank the support from the Institutional Foundation of the First Affiliated Hospital of Xi'an Jiaotong University (2021QN-05) and the National Natural Science Fund (No. 81700559).

## Conflict of Interest

The authors declare that the research was conducted in the absence of any commercial or financial relationships that could be construed as a potential conflict of interest.

## Publisher's Note

All claims expressed in this article are solely those of the authors and do not necessarily represent those of their affiliated organizations, or those of the publisher, the editors and the reviewers. Any product that may be evaluated in this article, or claim that may be made by its manufacturer, is not guaranteed or endorsed by the publisher.
